# A randomized controlled trial of isotonic versus hypotonic maintenance intravenous fluids in hospitalized children

**DOI:** 10.1186/1471-2431-11-82

**Published:** 2011-09-23

**Authors:** Thomas G Saba, James Fairbairn, Fiona Houghton, Diane Laforte, Bethany J Foster

**Affiliations:** 1 Dept. of Pediatrics, Montreal Children's Hospital, McGill University, Montreal, QC, Canada; 2 Faculty of Medicine, McGill University, Montreal, QC, Canada; 3 Dept. of Epidemiology, Biostatistics, and Occupational Health, McGill University, Montreal, QC, Canada

**Keywords:** hyponatremia, sodium, intravenous fluids, isotonic fluid

## Abstract

**Background:**

Isotonic saline has been proposed as a safer alternative to traditional hypotonic solutions for intravenous (IV) maintenance fluids to prevent hyponatremia. However, the optimal tonicity of maintenance intravenous fluids in hospitalized children has not been determined. The objective of this study was to estimate and compare the rates of change in serum sodium ([Na]) for patients administered either hypotonic or isotonic IV fluids for maintenance needs.

**Methods:**

This was a masked controlled trial. Randomization was stratified by admission type: medical patients and post-operative surgical patients, aged 3 months to 18 years, who required IV fluids for at least 8 hours. Patients were randomized to receive either 0.45% or 0.9% saline in 5.0% dextrose. Treating physicians used the study fluid for maintenance; infusion rate and the use of additional fluids were left to their discretion.

**Results:**

Sixteen children were randomized to 0.9% saline and 21 to 0.45% saline. Baseline characteristics, duration (average of 12 hours) and rate of study fluid infusion, and the volume of additional isotonic fluids given were similar for the two groups. [Na] increased significantly in the 0.9% group (+0.20 mmol/L/h [IQR +0.03, +0.4]; P = 0.02) and increased, but not significantly, in the 0.45% group (+0.08 mmol/L/h [IQR -0.15, +0.16]; P = 0.07). The rate of change and absolute change in serum [Na] did not differ significantly between groups.

**Conclusions:**

When administered at the appropriate maintenance rate and accompanied by adequate volume expansion with isotonic fluids, 0.45% saline did not result in a drop in serum sodium during the first 12 hours of fluid therapy in children without severe baseline hyponatremia. Confirmation in a larger study is strongly recommended.

**Clinical Trial Registration Number:**

NCT00457873 (http://www.clinicaltrials.gov/)

## Background

For almost half a century, pediatricians have ordered "maintenance" intravenous (IV) fluids for children according to the guidelines set out by Holliday and Segar: 100 cc/kg/day for the first 10 kg, plus 50 cc/kg/day for the next 10 kg, plus 20 cc/kg/day for each remaining kilogram [1]. Based on these recommendations for water intake, and on the estimated daily sodium and potassium needs of 3 milliequivalents and 2 milliequivalents per 100 kcal per day respectively, a hypotonic solution (0.2% saline) was recommended.

The wisdom of this approach to IV maintenance fluid therapy has been questioned recently [2-9]. Several authors have argued that administration of hypotonic fluids to hospitalized children - many of whom have a non-osmotic stimulus for anti-diuretic hormone (ADH) secretion - may lead to clinically important hyponatremia [10-13]. Iatrogenic hyponatremia has been the reported cause of neurological injury or death in more than 50 cases [13-18]. In many of these cases, fluids were administered at rates well above those typically recommended for maintenance [19]- a practice that has since been identified as a risk factor for hyponatremia [10]. The likelihood and severity of complications depend on both the rate of fall in serum sodium concentration [Na] and the absolute [Na] [20].

Concerns regarding hyponatremia have led some authors to recommend using isotonic saline as the routine maintenance solution for hospitalized patients, reserving hypotonic fluids for exceptional situations [9,19,21,22]. Isotonic saline administration may result in an increase in serum [Na] and/or chloride, [23-25] but has not been shown to increase the risk for hypernatremia [26]. Six randomized trials have demonstrated significantly greater drops in [Na] among patient receiving hypotonic solutions compared with those receiving isotonic solutions [25,27-31]. However, these trials may not be generalizable to the general population of children requiring maintenance IV fluids. Two studies were limited to critically ill children [27,29]. Two trials in surgical patients included the intra-operative period in addition to the immediate post-operative period [28,30]; the intraoperative period may be physiologically distinct from the post-operative period, and may be better considered separately. Two of these trials compared 0.9% saline with 0.18% saline. This solution is rarely used in practice today [27,31]. In fact the United Kingdom National Patient Safety Agency recommended removal of 0.18% saline from hospital stock in 2007, to reduce the risk of iatrogenic hyponatremia [32]. An additional trial focused on choice of fluid for volume expansion, rather than for maintenance needs: Neville compared 0.9% to 0.45% saline for rapid rehydration of children with gastroenteritis [25]. However, a fall in [Na] is a predictable outcome if hypotonic fluid is administered to volume depleted children. Hypotonic solutions are not recommended for volume expansion --only for maintenance; isotonic solutions are suggested for volume repletion [8,32].

Although the best choice of solution for IV maintenance needs has not yet been defined, many clinicians, including those at our institution, commonly prescribe 0.45% saline as a sort of 'compromise' between the traditional 0.18% saline and a complete switch to isotonic fluids. Therefore, we chose to compare 0.9% saline in 5.0% dextrose to 0.45% saline in 5.0% dextrose (subsequently referred to as 0.9% and 0.45% saline, respectively). We will refer to 0.9% saline in 5% dextrose as 'isotonic' and 0.45% saline in 5% dextrose as 'hypotonic', as per convention. It should be recognized that both of these solutions are in fact hypertonic to plasma. However, because dextrose is metabolized quickly after infusion, these solutions are effectively isotonic (0.9%) and hypotonic (0.45%). We hypothesized that serum [Na] would fall among children receiving 0.45% saline and remain stable or increase among children receiving isotonic fluids. The primary aim of this study was to estimate the rate of change in [Na] for patients administered each of hypotonic (0.45% saline) and isotonic (0.9% saline) IV fluids for maintenance needs, and to compare these rates. Rate of change in [Na] was selected as the primary outcome to allow 'standardization' of changes in [Na] to a fixed time interval (since the interval between baseline and exit [Na] was not the same for all participants). The absolute change in serum [Na], and the proportions with a decrease in [Na], hyponatremia ([Na] < 136 mmol/L), hypernatremia ([Na] > 145 mmol/L), hypertension, and adverse events related to hyponatremia were also considered.

## Methods

### Setting and Subjects

This prospective, double-blind, randomized controlled trial was conducted at the Montreal Children's Hospital, a tertiary care pediatric hospital. Two populations of children between 3 months and 18 years of age were recruited: children with medical illnesses admitted via the emergency department (medical), and children admitted following elective surgery (surgical). Only those requiring at least 8 hours of IV fluids were eligible. Exclusion criteria included a baseline serum [Na] of less than 133 or greater than 145 mmol/L, renal disease, cardiac dysfunction, pre-existing hypertension, diuretic use, edema, known adrenal dysfunction, and acute or severe chronic neurological illness. Children with neurological illnesses were excluded for safety reasons (high risk of non-physiologic ADH secretion, and difficulty in assessing changes in neurological status).

### Power considerations

A retrospective chart review of hospitalized children similar to those included in this trial indicated that the standard deviation for the rate of change in [Na] among patients administered hypotonic IV fluids was 0.29 mmol/L/h. This estimate was based, of necessity, on the change in [Na] observed among children in whom [Na] was measured at baseline and repeated at least once during IV fluid administration; both baseline and follow-up [Na] were obtained in only 20% of children receiving IV fluids. We planned to enroll 25 subjects per group, giving us 80% power to detect a difference of 0.21 mmol/L/h between groups with a one-sided test, setting alpha at 0.05. A one-sided test was used since it was felt to be physiologically very unlikely that the 0.9% saline group would experience a larger drop (or smaller rise) in [Na] than the 0.45% saline group. This number also gave us 80% power to detect a change of at least 0.17 mmol/L/hr in each group, with a two-sided test.

### Intervention

Eligible children were invited to participate if they were expected to receive maintenance IV fluids for at least 8 hours. Participants were randomized to receive either 0.9% saline in 5.0% dextrose or 0.45% saline in 5.0% dextrose. Solution bags were covered with an opaque plastic covering by the pharmacist. Participants, the treating team and the research team were blinded to the contents of the solution bags.

Randomization was stratified by admission type (medical vs. surgical) and carried out in blocks of six using a computerized random number generator. Baseline serum [Na] was drawn prior to starting study fluid. In medical patients, this was done when the IV catheter was inserted; surgical patients had their blood drawn upon completion of the surgical procedure. The rate of infusion of study fluid, use of fluid boluses or additional IV fluids, and addition of potassium to solutions were left to the discretion of the treating physicians. Treating physicians were informed that the study fluid was either 0.9% or 0.45% saline in 5% dextrose, and advised to prescribe it as they would normally prescribe maintenance fluids. Oral fluids were not restricted and were considered to be composed of free water. Serum [Na] was repeated approximately 12 hours (minimum 8; maximum 18 hours) after the beginning of the infusion, at which point the study ended. Serum [Na] was measured using indirect potentiometry using the Beckman Coulter DxC analyzer, which has a range of [Na] detection between 100-200 mmol/L and a coefficient of variation of 1%.

### Monitoring

All participants received routine in-patient nursing care, with vital signs including blood pressure monitored at least every 4 hours. Nurses also monitored for possible adverse events including symptoms potentially associated with hyponatremia (vomiting, change in level of consciousness, headache) or volume overload (edema, respiratory distress). All fluid intake during the study interval, including oral, was recorded.

### Statistical Analysis

The rate of change in serum [Na] was calculated as (exit [Na] - baseline [Na]) divided by the number of hours between baseline and exit blood draws. The rate of maintenance fluid administration was expressed as a percentage of the standard recommended rate [1]. The volume of electrolyte-free water received during the study interval was expressed as a percent of the total fluid volume received, as described previously [10].

Because the rate of change in [Na], and absolute change in [Na], were not normally distributed, the medians were compared using the Wilcoxon rank sum test, and difference from zero was tested using the Wilcoxon signed rank test. Categorical variables were compared with chi-square. A P-value < 0.05 was considered significant. All statistical analyses were performed using Stata/SE 10.0 (College Station, Texas).

### Ethics approval

The study was approved by the institutional Research Ethics Board. Written informed consent was obtained from parents or guardians for all participants and assent was obtained from children over 7 years of age if appropriate.

## Results

**Figure 1:**
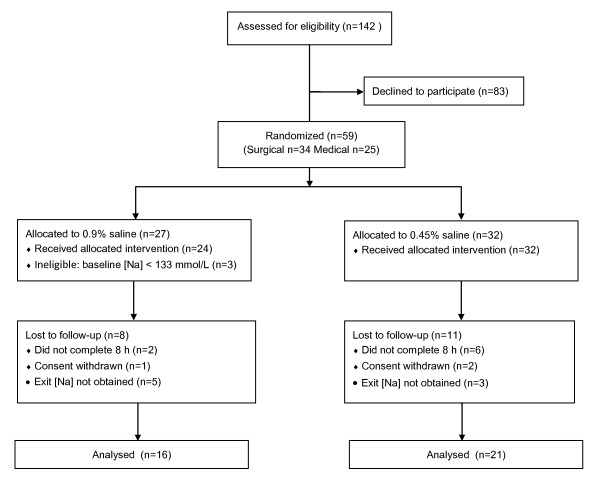
**Flow diagram of patients invited to participate**. For practical reasons it was necessary to randomize patients before eligibility was confirmed. Study fluid was often started while awaiting the results of the baseline [Na]-- which was used to determine eligibility. It was not always possible to predict which patients would require at least 8 hours of study fluid. Eight medical patients were withdrawn from the study (no exit [Na] obtained) when IV fluids were discontinued prior to completion of 8 hours of study fluid.

As illustrated in Figure 1, 12 of the 25 medical patients and 25 of the 34 surgical patients who enrolled completed the study. No exit [Na] was obtained for patients who did not complete.

### Participant characteristics

Characteristics of children receiving 0.9% saline (n = 16) were similar to those receiving 0.45% saline (n = 21) with respect to age, sex, diagnosis, and baseline [Na] (Table 1).

**Table 1:** Participant characteristics

	0.9% saline	0.45% saline
n	16	21

Age (y), median [IQR]	8.2 [2.8, 14.3]	8.9 [1.7, 16.5]

Males (n) (%)	8 (50)	10 (48)

Diagnosis		

Medical (total) (%)	6 (38)	6 (29)

Gastroenteritis	4 (25)	5 (24)

Pneumonia	1 (6)	1 (5)

Other	1 (6)	0 (0)

Surgical (total) (%)	10 (62)	15 (71)

Bowel surgery	2 (13)	3 (14)

Orthopedic surgery	5 (31)	8 (38)

Other	3 (19)	4 (19)

Baseline [Na] (mmol/L) median [IQR]	138 [135.5, 139]	137 [135,138]

IQR, interquartile range

Compared with participants who completed the study, children who refused participation, or who were excluded after randomization were younger (median age 4.2 (IQR 1.3, 10.9) years), more likely to be male (60%), and more likely to have a medical, rather than surgical, condition (57%).

### Fluids administered

The duration and rate of study fluid administration, as well as the volume of other isotonic fluids received during the study period were similar for the two treatment groups (Table 2).

**Table 2:** Fluids Administered and [Na] changes

	0.9% saline	0.45% saline	P-value*
**Fluid Administration (**median [IQR])			

Hours of study fluid administration	12 [10, 12.7]	11.9 [11.6, 12.3]	0.97

Rate (as % traditional maintenance rate)	105 [95,141]	104 [99,123]	0.80

Additional isotonic fluids (ml/kg)	7.4 [3.4, 18.6]	8.6 [2.8, 19.3]	0.84

Oral fluids (ml/kg)	4.7 [0.2, 8.3]	0 [0,6]	0.14

Free water (ml/kg)	4.7 [0.7, 8.3]	16.5 [12, 29.5]	0.00

Free water (% of total fluids received)	12 [1,20]	45 [37,64]	0.00

**[Na] Data **(median [IQR])			

Rate of change [Na] (mmol/L/hr)	+0.20 [0.03, 0.4]	+0.08 [-0.15, 0.16]	0.12

Absolute change in [Na] (mmol/L)	+3.0 [0.5, 4.5]	+1.0 [0.0, 2.0]	0.14

Exit [Na] (mmol/L)	140 [138,141]	138 [136,139]	0.10

**[Na] Data **(number (%))			

No. of patients with a fall in [Na] (%)	3 (19)	5 (24)	0.7

No. of patients with exit Na < 136 mmol/L (%)	1 (6)	1 (5)	1

*P-values for continuous variables were determined using 2-sided (Wilcoxon) rank sum tests

P-values for categorical variables were determined using Chi-square tests

The majority of participants received supplemental isotonic fluids in addition to the study fluid. Most of this supplemental fluid was administered as boluses, but some surgical patients received infusions of isotonic fluid in the interval between the baseline blood draw and start of study fluid.

### Changes in serum [Na]

On average, [Na] increased in both treatment groups (Table 2). However, the rate of change in [Na] was significantly different from zero only for the 0.9% group (+0.20 mmol/L/h [IQR 0.03, 0.4]; P = 0.02). For the 0.45% group, [Na] increased by +0.08 mmol/L/h [IQR -0.15, 0.16], which was not significantly different from zero (P = 0.07). The rate of change in [Na] did not differ significantly between treatment groups. The absolute change in [Na] was greater for the 0.9% saline group (+3.0 mmol/L [IQR 0.5, 4.5]) compared with the 0.45% saline group (+1.0 mmol/L [IQR 0.0, 2.0]), however, this difference was not statistically significant.

**Figure 2:**
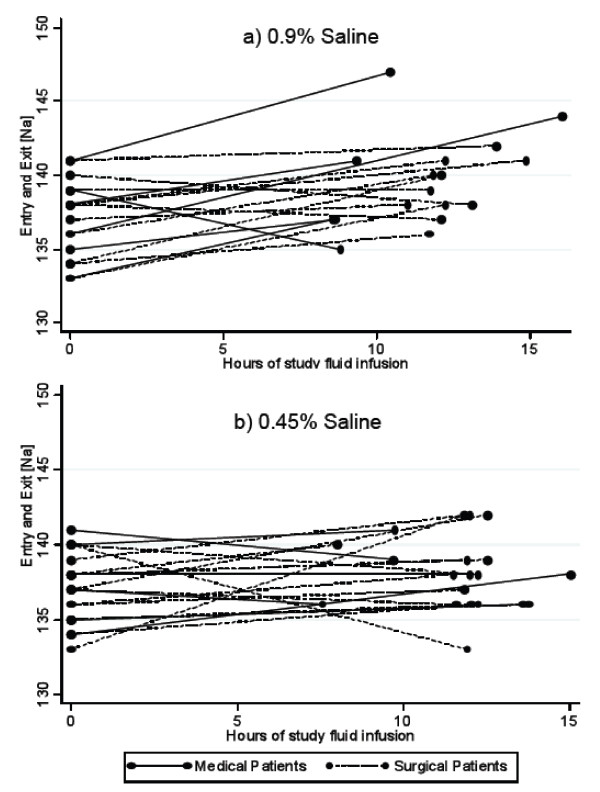
**Changes in serum [Na] in patients receiving 0.9% saline (a) and 0.45% saline (b)**. Each line represents an individual patient.

As illustrated in Figure 2, there was variability among participants with respect to both direction and rate of change in [Na]. Twenty-four percent (5/21) of the patients in the 0.45% saline group experienced a drop in serum [Na], compared with 19% (3/16) of those in the 0.9% saline group (p = 0.7). The lowest exit [Na] (133 mmol/L) and maximum rate of fall (-0.52 mmol/L/h) were in a surgical patient receiving 0.45% saline; this patient had 380 ml of oral fluid intake during the study interval. The second lowest exit [Na] (135 mmol/L) and next fastest rate of fall (-0.40 mmol/L/h) were in a medical patient receiving 0.9% saline; oral intake was 82 ml.

There were no adverse events. No participant developed hypertension. A 6 year-old boy with gastroenteritis in the 0.9% saline group developed mild hypernatremia, with an exit [Na] of 147 mmol/L.

### Stratified analysis

Fluid management differed between medical and surgical patients. Therefore, although numbers were small, an exploratory analysis, stratified by admission type, was also conducted. Medical patients received the study fluid at 142% [IQR 118, 150] of the traditional maintenance rate, and 19.7 ml/kg [IQR 14.7, 20.8] of additional isotonic fluids, mostly in the form of boluses. In contrast, surgical patients received study fluid at 101% [IQR 94, 108] of the traditional maintenance rate, and received only 3.4 ml/kg [IQR 2.6, 8.7] of additional isotonic fluids. The duration and rate of study fluid administration, and volume of additional isotonic fluids were similar for the 0.9% and 0.45% saline groups overall. The changes in [Na] observed when medical and surgical patients were analyzed separately mirrored those in the unstratified analysis.

## Discussion

Contrary to our expectations, we observed increases in serum sodium in both the 0.45% and 0.9% groups, although this change was only statistically significant in the 0.9% group. Furthermore, similar proportions of individuals in both treatment arms developed hyponatremia.

After decades of using hypotonic fluids, the best choice of solution for maintenance IV fluid therapy has recently become a topic of intense debate. Six randomized trials favored isotonic over hypotonic fluids for the prevention of hyponatremia [25,27-31]. There were two main differences between the present trial and past studies. First, the present study included a more diverse population than past trials, including both non-critically ill general medical patients and surgical patients after completion of surgery. Second, prior trials dictated both the rate and composition of fluids administered. A strength of the present study is that only fluid *composition *was dictated by study protocol; physicians were advised that study fluid was to be used for maintenance needs, and allowed the freedom to choose the rate of administration and any additional fluids according to their usual practice. This allowed us to isolate the impact of fluid composition on changes in serum [Na] in a 'real world' clinical setting in which physicians determined the rate of fluid administration.

Maintenance fluids are one of the three key components of an IV fluid prescription, along with deficit replacement, and replacement of ongoing losses [33]. The goal of maintenance fluids is to maintain fluid homeostasis by replacing both insensible water losses and obligate urinary water losses. Combining theoretical considerations regarding obligate urinary water losses, empiric estimates of average daily insensible water losses, and estimates of daily electrolyte requirements, Holliday and Segar recommended hypotonic solutions (0.2% saline) for the maintenance component of the IV fluid prescription [1]. However, their assumptions regarding obligate urinary water losses - or at least regulation of urinary water losses -may not be valid among hospitalized children.

Recommendations for replacement of a volume deficit vary. For rapid volume expansion, only isotonic fluids are considered safe and effective [34]. Although some sources suggest that 0.45% saline can be used for slow correction of volume deficits [35], use of 0.45% saline in a volume depleted patient (who therefore has elevated ADH) will predictably lead to a drop in serum [Na]. Volume deficits are isotonic deficits, so should be replaced with isotonic fluid. This view is reflected in current recommendations [32,34]. The composition of fluid used for ongoing losses depends on the type of fluid being lost.

Regardless of the recommendation to consider deficit replacement separately from maintenance fluid needs, it has been common practice to simply increase the rate of infusion of the maintenance fluid solution to "1.5 times or 2 times maintenance" in an effort to replace volume deficits that remain after bolus isotonic fluids have been given for rapid intravascular volume expansion. This practice was evident among the medical patients participating in the present study, who received study fluid at an average of 142% of the traditional maintenance rate. Our study design isolated the impact of differing maintenance fluid composition on [Na] in the context of current prescription practices.

As expected, we observed a significant increase in serum [Na] among patients in the 0.9% saline group. A smaller and slower, though not statistically significant, increase in [Na] was observed in the 0.45% saline group. This was not anticipated. Rather, we had hypothesized that patients randomized to receive hypotonic fluids would experience a drop in serum [Na]. The rationale for this hypothesis was that the patients enrolled in the study were at risk for high ADH secretion, stimulated by either volume depletion (appropriately) or the syndrome of inappropriate ADH secretion (due to pain, medications, pulmonary disease, etc.). Because free water excretion is impaired in the presence of ADH, the administration of hypotonic solutions to patients secreting ADH will inevitably lead to a fall in serum [Na] [10].

The fact that most children who received 0.45% saline did not experience a drop in [Na] suggests that most did not have an ongoing stimulus for ADH secretion. Adequate volume repletion with isotonic fluids prior to and during IV maintenance fluid administration likely protected patients against hyponatremia despite hypotonic maintenance fluids. Those who did experience a drop in [Na] can be assumed either to have not been adequately volume expanded (and therefore have ongoing physiologic volume-related stimulus for ADH secretion) or to have had inappropriate ADH secretion (non-physiologic, unpredictable ADH secretion). Children in the 0.9% saline group who experienced a decrease in [Na] were very likely to have had inappropriate ADH secretion; unless they developed new onset adrenal insufficiency, hypothyroidism, or renal salt wasting, inappropriate ADH secretion is the only reasonable explanation for a drop in [Na] in this group [25,36].

The average rate of study fluid administration in both treatment groups was only slightly greater than the traditionally recommended maintenance rate. This likely also played a role in the relative stability in serum [Na]. Hypotonic fluids prescribed at high rates have been implicated in many of the reported deaths due to iatrogenic hyponatremia [19].

Two features of the study design may have resulted in bias toward finding no difference between treatment groups. First, the exclusion for safety reasons of children with baseline [Na] < 133 mmol/L or serious neurological disease may have eliminated those children at the highest risk for progressive hyponatremia. Second, this study focused on the first 12 hours of maintenance fluids. This was done in part for practical reasons, but also because we hypothesized that more pain and a greater degree of volume depletion would make this early period the period of highest risk for non-osmotic stimuli for ADH secretion. However, the syndrome of inappropriate ADH secretion may occur at any time, and may be more likely to occur later in the hospitalization, particularly in surgical patients [10,27]. If the risk of inappropriately elevated ADH increases with increasing duration of hospitalization, then evaluating only the first 12 hours of IV fluids may have biased towards finding no difference between the groups.

This was a small trial. The small sample limited our ability to detect small differences between the two treatment groups. It is possible that both the observed difference between treatment groups and the change in [Na] in the 0.45% saline group would have been statistically significant with a larger sample. The relatively large proportion of patients who failed to complete the study was an additional limitation. There were some differences in the characteristics of patients who completed the study compared with those who did not. This may limit generalizability of findings.

It is also important to recognize that this study was designed to examine intermediate outcomes (rate of change in [Na], absolute change in [Na], exit [Na]) rather than a clinically important outcome (complications associated with choice of fluid). This was done for power reasons: clinical complications are rare and would require a very large sample. Complications of hyponatremia are believed to depend on both the magnitude of the serum [Na] and the rate of change; therefore, these were considered relevant outcomes. However, much larger trials, examining clinically important outcomes, are required to establish the safety of different maintenance fluids.

The most important finding of this study was that there was no decrease in [Na] in the 0.45% saline group; this can likely be attributed to judicious use of volume expansion with isotonic fluids and to the fact that, in most patients, study fluids were prescribed at rates generally not exceeding traditional maintenance rates. This result highlights the importance of considering maintenance fluids separately from deficit replacement.

It is important to recognize that there is no IV fluid strategy that will completely eliminate the risk of iatrogenic hyponatremia. Hypotonic fluids in the setting of subtle, unrecognized volume depletion will result in a drop in [Na]. In the setting of the syndrome of inappropriate ADH secretion, [Na] may fall even with isotonic fluids - although larger drops will occur with hypotonic fluids. The use of isotonic fluids may reduce the risk of important hyponatremia, but if tragic consequences of IV fluids are to be avoided, monitoring of serum [Na] is important.

## Conclusion

Firm conclusions regarding the best solution for maintenance IV fluids cannot be drawn from this small study. Larger studies, powered to detect differences in the incidence of clinically important adverse events such as symptomatic hyponatremia or hypernatremia and hypertension, in addition to differences in change in [Na], are needed. In addition, studies of longer duration would be useful. This study suggests that 0.45% saline does not result in a drop in [Na] during the first 12 hours of fluid therapy among children with a baseline [Na] > 133 mmol/L and < 145 mmol/L, if appropriate care is taken to restore volume deficits with isotonic fluids, and excessive fluid administration rates are avoided. However, confirmation of these results in a larger study is strongly recommended.

## Abbreviations

The following abbreviations appear: ADH: antidiuretic hormone; IQR: interquartile range; IV: intravenous; Na: sodium.

## Competing interests

The authors declare that they have no competing interests.

## Authors' contributions

TS was involved in patient recruitment, data collection, data synthesis, statistical analysis and drafted the manuscript; JF helped in the chart review and data entry; DL and FH recruited patients; BF conceived of the study, oversaw the entire project, and edited the manuscript. All authors have read and approved this manuscript.

## Pre-publication history

The pre-publication history for this paper can be accessed here:

http://www.biomedcentral.com/1471-2431/11/82/prepub
